# Clinical and volumetric changes with increasing functional impairment in familial frontotemporal lobar degeneration

**DOI:** 10.1016/j.jalz.2019.08.196

**Published:** 2020-01-06

**Authors:** Nicholas T. Olney, Elise Ong, Sheng-Yang M. Goh, Lynn Bajorek, Reilly Dever, Adam M. Staffaroni, Yann Cobigo, Meredith Bock, Kevin Chiang, Peter Ljubenkov, John Kornak, Hilary W. Heuer, Ping Wang, Katya Rascovsky, Amelia Wolf, Brian Appleby, Jessica Bove, Yvette Bordelon, Patrick Brannelly, Danielle Brushaber, Christine Caso, Giovanni Coppola, Bradford C. Dickerson, Susan Dickinson, Kimiko Domoto-Reilly, Kelly Faber, Jessica Ferrall, Julie Fields, Ann Fishman, Jamie Fong, Tatiana Foroud, Leah K. Forsberg, Debra J. Gearhart, Behnaz Ghazanfari, Nupur Ghoshal, Jill Goldman, Jonathan Graff-Radford, Neill R. Graff-Radford, Ian Grant, Murray Grossman, Dana Haley, Gingyuek Hsiung, Edward D. Huey, David J. Irwin, David T. Jones, Kejal Kantarci, Anna M. Karydas, Daniel Kaufer, Diana Kerwin, David S. Knopman, Joel H. Kramer, Ruth Kraft, Walter Kremers, Walter Kukull, Maria I. Lapid, Irene Litvan, Ian R. Mackenzie, Miranda Maldonado, Masood Manoochehri, Scott M. McGinnis, Emily C. McKinley, Mario F. Mendez, Bruce L. Miller, Chiadi Onyike, Alex Pantelyat, Rodney Pearlman, Len Petrucelli, Madeleine Potter, Rosa Rademakers, Eliana M. Ramos, Katherine P. Rankin, Erik D. Roberson, Emily Rogalski, Pheth Sengdy, Leslie M. Shaw, Jeremy Syrjanen, M. Carmela Tartaglia, Nadine Tatton, Joanne Taylor, Arthur Toga, John Q. Trojanowski, Sandra Weintraub, Bonnie Wong, Zbigniew Wszolek, Adam L. Boxer, Brad F. Boeve, Howard J. Rosen

**Affiliations:** a Department of Neurology, Memory and Aging Center, University of California, San Francisco, San Francisco, CA, USA; b Department of Neurology, Perelman School of Medicine, University of Pennsylvania, Philadelphia, PA, USA; c Department of Neurology, Case Western Reserve University, Cleveland, OH, USA; d Department of Neurology, University of California, Los Angeles, Los Angeles, CA, USA; e Tau Consortium, Rainwater Charitable Foundation, Fort Worth, TX, USA; f Department of Health Sciences Research, Mayo Clinic, Rochester, MN, USA; g Department of Neurology, University of Washington, Seattle, WA, USA; h Department of Neurology, Frontotemporal Disorders Unit, Massachusetts General Hospital, Harvard Medical School, Boston MA, USA; i Association for Frontotemporal Degeneration, Radnor, PA, USA; j National Centralized Repository for Alzheimer’s Disease and Related Disorders (NCRAD), Indiana University, Indianapolis, IN, USA; k Department of Neurology, University of North Carolina, Chapel Hill, NC, USA; l Department of Neurology, Mayo Clinic, Rochester, MN, USA; m Department of Psychiatry and Behavioral Sciences, School of Medicine, Johns Hopkins University, Baltimore, MD, USA; n Division of Neurology, Department of Medicine, University of Toronto, Toronto, Ontario, Canada; o Department of Psychiatry, Washington University, St. Louis, MO, USA; p Department of Neurology, Washington University, St. Louis, MO, USA; q Taub Institute for Research on Alzheimer’s Disease and the Aging Brain, Columbia University, New York, NY, USA; r Department of Neurology, Columbia University, New York, NY, USA; s Department of Neurology, Mayo Clinic, Jacksonville, FL, USA; t Department of Neurology, Feinberg School of Medicine, Northwestern University, Chicago, IL, USA; u Division of Neurology, Department of Medicine, University of British Columbia, Vancouver, British Columbia, Canada; v Department of Neurology and Neurotherapeutics, Center for Alzheimer’s and Neurodegenerative Diseases, The University of Texas, Southwestern Medical Center at Dallas, Dallas, TX, USA; w Department of Internal Medicine, The University of Texas, Southwestern Medical Center at Dallas, Dallas, TX, USA; x National Alzheimer Coordinating Center (NACC), University of Washington, Seattle, WA, USA; y Department of Neurosciences, Parkinson and Other Movement Disorders Center, University of California, San Diego, San Diego, CA, USA; z Department of Pathology and Laboratory Medicine, University of British Columbia, Vancouver, BC, Canada; aa Department of Neurology, Alzheimer’s Disease Center, University of Alabama at Birmingham, Birmingham, AL, USA; bb Department of Neurology, School of Medicine, Johns Hopkins University, Baltimore, MD, USA; cc The Bluefield Project, San Francisco, CA, USA; dd Tanz Centre for Research in Neurodegenerative Diseases, University of Toronto, Toronto, ON, Canada; ee Laboratory of Neuroimaging (LONI), University of Southern California, Los Angeles, CA, USA

**Keywords:** Familial, Genetic, Frontotemporal lobar degeneration, *MAPT*, *GRN*, *C9ORF72*

## Abstract

**Introduction:**

The Advancing Research and Treatment in Frontotemporal Lobar Degeneration and Longitudinal Evaluation of Familial Frontotemporal Dementia Subjects longitudinal studies were designed to describe the natural history of familial-frontotemporal lobar degeneration due to autosomal dominant mutations.

**Methods:**

We examined cognitive performance, behavioral ratings, and brain volumes from the first time point in 320 *MAPT*, *GRN*, and *C9orf72* family members, including 102 non–mutation carriers, 103 asymptomatic carriers, 43 mildly/questionably symptomatic carriers, and 72 carriers with dementia.

**Results:**

Asymptomatic carriers showed similar scores on all clinical measures compared with noncarriers but reduced frontal and temporal volumes. Those with mild/questionable impairment showed decreased verbal recall, fluency, and Trail Making Test performance and impaired mood and self-monitoring. Dementia was associated with impairment in all measures. All *MAPT* carriers with dementia showed temporal atrophy, but otherwise, there was no single cognitive test or brain region that was abnormal in all subjects.

**Discussion:**

Imaging changes appear to precede clinical changes in familial-frontotemporal lobar degeneration, but specific early clinical and imaging changes vary across individuals.

## 1. Introduction

Frontotemporal lobar degeneration (FTLD) is a progressive, currently incurable, neurodegenerative disease that is most commonly associated with central nervous system accumulation of one of two proteins: tau or transactive response DNA-binding protein 43 [1]. Most efforts to develop treatments for FTLD are focusing on clearing and/or decreasing formation of these proteins [2]. Studies of such treatments will be more challenging because of the clinical heterogeneity of FTLD, which can present with a variety of syndromes [3]. Increasing evidence indicates that prediction of the specific FTLD protein based on the clinical syndrome can be unreliable [3]. This problem has fueled interest in cohorts of patients with FTLD in whom the protein pathology is predictable.

Up to 40% of FTLD cases present as a dominantly inherited familial disorder (f-FTLD). Mutations in three genes account for over 50% of f-FTLD: microtubule-associated tau (*MAPT*), progranulin (*GRN*), and chromosome 9 open reading frame 72 (*C9orf72*). Treatment studies in f-FTLD are particularly important because each mutation is highly predictive of a specific proteinopathy [4]. In addition, because f-FTLD participants can be identified before symptoms begin, studies can evaluate the effect of a treatment in the earliest phases of illness and also test whether a treatment delays or prevents onset of symptoms.

These considerations led to the creation of the Longitudinal Evaluation of Familial Frontotemporal Dementia Subjects (LEFFTDS) and Advancing Research and Treatment in Frontotemporal Lobar Degeneration (ARTFL) studies, which were designed to understand the natural history of f-FTLD by longitudinally following up both symptomatic and asymptomatic mutation carriers. To maximize generalizability of the findings, the studies are mostly focusing on families with mutations in the genes most commonly associated with f-FTLD: *MAPT*, *GRN*, and *C9orf72*.

The current analysis presents data collected at the first time point from this cohort. We compared cognitive performance, behavioral ratings, and brain volumes across groups of asymptomatic and symptomatic carriers to identify the measures that might mark the early development of symptoms. One of the problems with group analysis, however, is that the findings may not apply to all individuals. This is a critical issue in f-FTLD, where each mutation affects the brain differently, and a person with a given mutation can present with a variety of symptoms [1]. Relying on a single test for all carriers may delay recognition of oncoming symptoms. To examine this issue, we quantified the frequency in which participants in each group showed abnormal performance in each cognitive measure and brain region.

## 2. Methods

Participants were recruited at one of 18 centers that are part of the ARTFL (https://www.rarediseasesnetwork.org/cms/artfl) and/or LEFFTDS (https://clinicaltrials.gov/show/NCT02372773) networks and included in this analysis if there was a confirmed mutation in the *MAPT*, *GRN*, or *C9orf72* genes in at least one family member. Clinicians were blinded to each participant’s mutation status unless the participant had learned their mutation status.

### 2.1. Clinical assessment

Participants had a uniform multidisciplinary assessment that includes neurological history and examination, collateral source interview, and neuropsychological testing. Most of the clinical measures come from the third version of the NIH National Alzheimer’s Coordinating Center’s (NACC) Uniform Data Set neuropsychological battery ([5]; www.alz.washington.edu), which includes a module for assessment of FTLD. The Uniform Data Set neuropsychological battery neuropsychological tasks included the Montreal Cognitive Assessment (MoCA), measures of verbal episodic memory (the Craft story recall task, which is similar to the Wechsler Memory Scale logical memory task), visual episodic memory (ten-minute recall for the Benson complex figure), visuospatial function (copy of the Benson figure), naming (the Multilingual Naming Test [MINT]), lexical fluency (generation of words beginning with the letters “F” and “L”, each in one minute), category fluency (generation of animal and vegetable names, each in one minute), attention (forward digit span, Trail Making Test part A), working memory (backward digit span), and set shifting (Trail Making Test part B). Additional tasks included the short form of the California Verbal Learning Test [6]. Measures to characterize socioemotional behavior included the short version of the Neuropsychiatric Inventory (NPI-Q [7]), the Revised Self-Monitoring Scale (RSMS [8]), and the Behavioral Inhibition Scale [9]. Mood was quantified with the Geriatric Depression Scale (GDS [10]). Motor function was quantified with the Unified Parkinson’s Disease Rating Scale [11] motor examination. General functional state was characterized using an expanded version of the Clinical Dementia Rating Scale (which is now known as the CDR^®^ Staging Instrument and will be abbreviated as CDR^®^ hereafter [12]). The CDR^®^ provides a categorical rating of severity in six domains, with scores ranging from 0 (clinically normal) to 0.5 (mild/questionable symptoms not affecting daily function) and to levels 1, 2, or 3 (all indicating significant impairment consistent with dementia) for each domain. To broaden the utility of the CDR^®^ into FTLD spectrum disorders, behavior/comportment/personality, and language domains have been added to the CDR^®^ to form the 8-domain “FTLD-CDR” [13], and these additional behavior and language domain ratings are implemented by the NACC. This 8-domain rating is now abbreviated as the “CDR^®^ plus NACC FTLD”. The Progressive Supranuclear Palsy Rating Scale [14] quantifies a combination of motor, behavior, and cognitive features relevant to progressive supranuclear palsy.

### 2.2. Genetic testing

Each participant had genetic testing to identify the presence or absence of specific mutations associated with FTLD. Details of the procedures and results of genetic testing are described in a separate publication (Ramos et al., this issue). Although all participants are offered the opportunity to undergo clinical genetic testing, most of the asymptomatic persons have chosen to refrain from clinical testing thus far. However, each participant undergoes research genetic testing (to which the clinicians remain blind and the results are not shared with participants), and therefore, the mutation status is determined for each participant.

### 2.3. Image acquisition

Participants were scanned on 3 Tesla MRI scanners from one of three vendors: Philips Medical Systems, Siemens, or General Electric Medical Systems. A standard imaging protocol was used, managed, and reviewed for quality by a core group at the Mayo Clinic, Rochester. The current analysis used the T1 weighted images, which were acquired as magnetization prepared rapid gradient echo images using the following parameters: 240×256×256 matrix; about 170 slices; voxel size = 1.05×1.05×1.25 mm^3^; flip angle, echo time and repetition time varied by vendor.

### 2.4. Image processing

Image processing was accomplished using SPM12 (http://www.fil.ion.ucl.ac.uk/spm) and previously published procedures [15]. Magnetic resonance imaging (MRI) scans were processed to create individualized voxel-wise maps quantifying the degree of atrophy for each individual. Volume loss at each voxel was quantified as a w-score, which represents the gray matter content at that voxel as the number of standard deviations away from the expected mean for a cognitively normal reference group after accounting for age, total intracranial volume, and scanner platform [16]. Reference images for creation of atrophy maps were obtained from 270 control subjects, including 115 noncarrier family members from ARTFL/LEFFTDS, 63 who enrolled in prior studies of neuroimaging in FTLD at University of California San Francisco (AG032306 [17]), 34 from non–mutation carriers from the Dominantly Inherited Alzheimer’s Network (; dian.wustl.edu), and 72 who participated in the Parkinson’s Progression Markers Initiative (; www.ppmi-info.org).

Cortical volumes for the frontal and temporal lobes for each individual were also calculated by transforming a brain parcellation atlas [18] into the study-specific brain space and summing all modulated gray matter within the frontal and temporal lobes. Peak coordinates for imaging findings are provided in the coordinates of the International Consortium for Brain Mapping brain template [19].

Additional details on the acquisition, quality control, and image-processing procedures are provided in the Supplementary Materials.

### 2.5. Creation of groups for analysis

The group was divided into four categories based on mutation status and clinical severity, as measured by the CDR^®^ plus NACC FTLD. The groups were asymptomatic non–mutation carriers (−mFTLD-CDR = 0), asymptomatic mutation carriers (+mFTLD-CDR = 0), mildly/questionably symptomatic mutation carriers (+mFTLD-CDR = 0.5), and symptomatic mutation carriers (+mFTLD-CDR ≥ 1). Consistent with the established approach for assigning these ratings, clinicians used a combination of direct patient observation and informant report to categorize each patient, and there was no formal incorporation of neuropsychological data. Because the CDR^®^ does not include categories for language and behavior, there is no established algorithm for creating an overall rating that includes the outcomes of these additional ratings. Consequently, patients may have subtle impairment due to language or behavioral problems and still be rated as 0 on the CDR^®^. Therefore, we created an algorithm to integrate ratings for all eight categories into a global rating for each individual. The rules were as follows:

1. If all domains are 0, the global CDR^®^ plus NACC FTLD score is 0.
2. If the maximum domain score is 0.5, the global CDR^®^ plus NACC FTLD score is 0.5.
3. If the maximum domain score is above 0.5 in any domain, then the following applies:
  - If the maximum domain score is 1 and all other domains are 0, the global CDR^®^ plus NACC FTLD score is 0.5.
  - If the maximum domain score is 2 or 3 and all other domains are 0, the global CDR^®^ plus NACC FTLD score is 1.
  - If the maximum domain score occurs only once and there is another rating besides zero, the global CDR^®^ plus NACC FTLD score is one level lower than the level corresponding to maximum impairment (e.g., if maximum = 2 and there is another rating besides zero, the global CDR^®^ plus NACC FTLD score is 1; if maximum = 1 and there is another rating besides zero, the global CDR^®^ plus NACC FTLD score is 0.5).
  - If the maximum domain score occurs more than once (e.g., 1 in 2 domains, 2 in 2 domains), then the global CDR^®^ plus NACC FTLD score is that maximum domain score.

### 2.6. Group comparisons

Changes occurring with disease stage were examined by comparing the mean value across groups for all clinical variables and for the frontal and temporal lobes using linear regression, treating each variable as an outcome and disease stage as a categorical predictor, and including age, sex, and education as covariates. For models where the effect of group was statistically significant (*P* < .05), we conducted targeted post-hoc analyses by comparing each mutation carrier group with the −mFTLD-CDR = 0 group as well as with the lower stages of disease (e.g., +mFTLD-CDR ≥ 1 was compared with −mFTLD-CDR = 0 and +mFTLD-CDR = 0.5). To maximize statistical power, these analyses were performed with all three types of mutations together. Statistical analysis was performed using R (www.R-project.org).

### 2.7. Consistency of abnormalities across individuals

One of the intended uses of these measures would be to indicate that a previously healthy mutation carrier is entering a new phase of illness where function is beginning to be affected. While changes in mean values with disease stage are informative for understanding which measures might mark these transitions, it is also important to understand how well these group observations apply to each individual. One way to examine this is to quantify the proportion of individuals that show abnormalities in each variable at each stage. The ARTFL/LEFFTDS team recently implemented a procedure for transforming each individual’s neuropsychological scores into age- and education-corrected standardized scores based on the normative data provided by the NACC. The details of the procedure are published elsewhere [20], and the procedure has not been implemented for all variables, but for those that have these transformations available, we examined the percent of individuals at each stage that were abnormal using a cutoff of z = −1.5. We took a similar approach with the imaging data by creating maps showing the proportion of individuals that had w-scores lower than −1.5 at every voxel. For these analyses, the data are presented separately for each mutation type to provide information about variability in specific symptoms across mutation types.

## 3. Results

Data were available for 320 individuals whose genotyping had been completed. They fell into the planned groups as follows: asymptomatic non–mutation carriers (−mFTLD-CDR = 0, n = 102), asymptomatic mutation carriers (+mFTLD-CDR = 0, n = 103), mildly/questionably symptomatic mutation carriers (+mFTLD-CDR = 0.5, n = 43), and overtly symptomatic mutation carriers (+mFTLD-CDR ≥ 1: n = 72). Demographics for each group are shown in Table 1.

### 3.1. Mean values across levels of severity

Linear models grouped by levels of severity combined across mutation carriers revealed statistically significant effects of group for nearly every variable examined (Table 1). Post-hoc testing revealed that this was largely driven by the +mFTLD-CDR ≥ 1 group, which showed significant impairments in all clinical variables and decreased frontal and temporal brain volumes compared with the −mFTLD-CDR = 0, +mFTLD-CDR = 0, and +mFTLD-CDR = 0.5 groups. The +mFTLD-CDR = 0.5 group showed significant differences on the MoCA, Craft Delayed Recall, California Verbal Learning Test-Delay, Benson-Delay, vegetable fluency, trails A and B, NPI-Q, GDS, and RSMS, on frontal and temporal volumes compared with the −mFTLD-CDR = 0 group, and decreases in vegetable fluency, “F” word fluency, NPI-Q, GDS, and RSMS compared with the +mFTLD-CDR = 0 group. In the +mFTLD-CDR = 0 group, there were no clinical variables that were significantly different compared with the −mFTLD-CDR = 0 group, but frontal and temporal volumes were statistically significantly decreased in the +mFTLD-CDR = 0 group. T-scores and more precise *P* values for these comparisons are provided in Supplementary Table 1 in the Supplementary Materials.

### 3.2. Frequency of impairment on cognitive testing

**Fig. 1.**
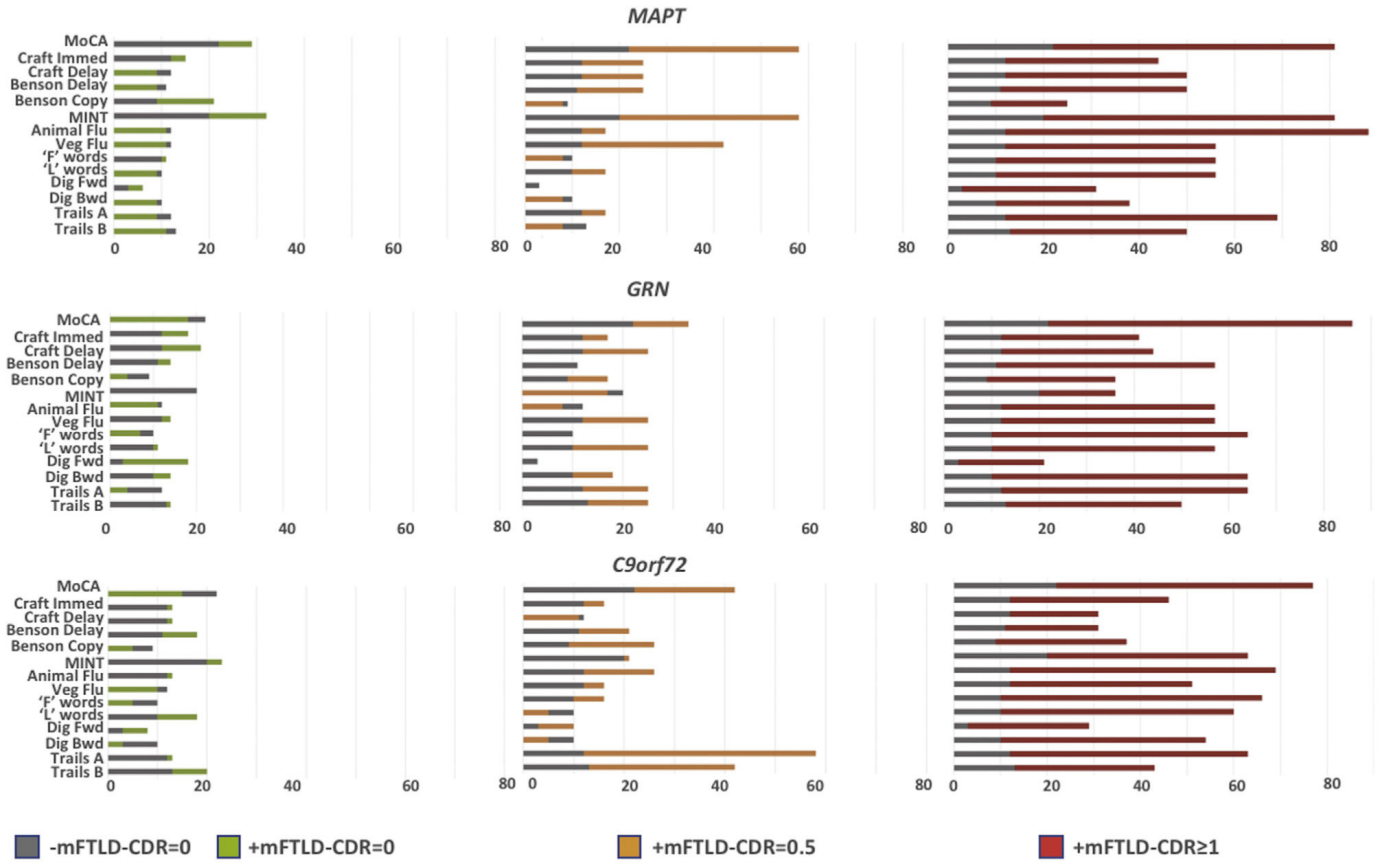
Proportion of individuals in each group with abnormal performance (z < −1.5) on each cognitive test with available norms (colored bars) superimposed on proportion of noncarriers with abnormal performance on that test. Bars extend to indicate largest observed proportion, so that bars where colors extend beyond gray indicate that mutation carrier group showed higher proportion (denoted by rightward extent of colored bar from the y-axis line) than noncarriers (whose proportion is denoted by rightward extent of gray bars from y-axis line). Abbreviations: MoCA, Montreal Cognitive Assessment; MINT, Multilingual Naming Test; MAPT, microtubule associated tau; GRN, progranulin; C9orf72, chromosome 9 open reading frame 72.

Data on the percentage of participants showing impairment in each cognitive test are shown in Fig. 1, with data for each mutation type and level of severity plotted in colored bars relative to the proportion of −mFTLD-CDR = 0 showing abnormality in that measure, plotted in gray bars. Additional details are shown in Supplementary Tables 2-5 in the Supplementary Materials including how many in each group had any abnormal test, how many had abnormal performance for each test, and, for each test, how many had abnormal performance on only that test. Seventy percent of individuals in the −mFTLD-CDR = 0 group showed abnormal performance for at least one score, with the most commonly abnormal test being the MoCA (22%; Fig. 1, gray bars; Supplementary Table 2), and the second most common being the MINT (20%).

For each mutation, abnormalities were sometimes more common in carriers compared with noncarriers in the FTLD-CDR = 0 stage, but the frequency of abnormalities increased along with overall disease severity (Fig. 1). For instance, the MoCA was abnormal in 22% of the −mFTLD-CDR = 0 group, and abnormal MoCA scores were more frequent in +mFTLD-CDR = 0 *MAPT* carriers, at 29% but less common in +mFTLD-CDR = 0 carriers of *GRN* (18%) and *C9orf72* (15%). Overall, about 70% to 80% of +mFTLD-CDR = 0 and +mFTLD-CDR = 0.5 carriers had at least one abnormal test, whereas nearly 100% had at least one abnormal test in the +mFTLD-CDR ≥ 1 group (Supplementary Tables 3-5). The MoCA was a commonly abnormal test (most common or second most common in nearly all groups), and the MINT was frequently abnormal. In particular, the MINT was the most common or second most commonly abnormal test at each level of severity in *MAPT* carriers, who had the most consistent pattern of abnormalities across levels of severity (Fig. 1; Supplementary Table 3). Among *GRN* carriers, abnormal performance on the Craft story recall task was relatively common, along with Trail Making Test and “F” word fluency (Fig. 1, Supplementary Table 4). In *C9orf72* carriers, there appeared to be the least consistency across levels of severity beyond the MoCA (Fig. 1, Supplementary Table 5). There was no group in whom the same test was abnormal in 100% of participants, and in all mutation types, there was a substantial number of individuals who had only one abnormal test that was not the most common test. For instance, in the +mFTLD-CDR = 0 *C9orf72* group (Supplementary Table 5), the most common abnormal task was the MINT (9 people, 23% of participants), but 20 (50% of people) performed normally on the MINT but abnormally on another task and 12 people (30%) were abnormal on only one test that was not the MINT.

### 3.3. Regional volume loss across individuals

**Fig. 2.**
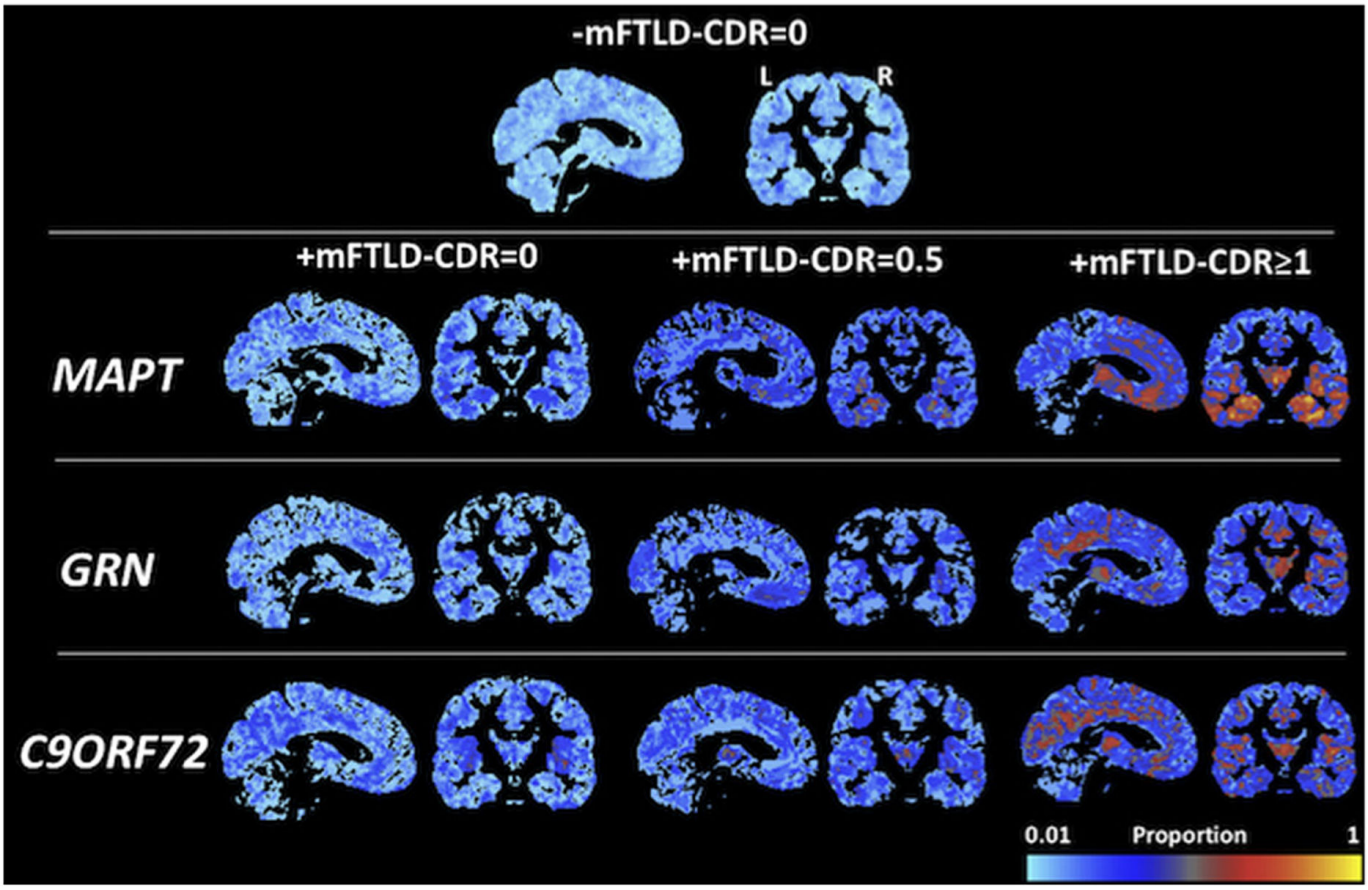
Proportion of individuals in each group with reduced gray matter volume (w-score < −1.5) at each gray matter voxel. Increasing color from blue to yellow in “heat map” indicates higher proportion of individuals in that group showed reduced volume at that location. Left hemisphere is displayed on the left in coronal images. Abbreviations: MAPT, microtubule associated tau; GRN, progranulin; C9orf72, chromosome 9 open reading frame 72.

In every group, there was at least one voxel that was more than 1.5 w-score units below normal (Fig. 2). In the −mFTLD-CDR = 0 group, the maximum proportion of individuals with abnormal gray matter at any voxel reached about 0.3. In the *MAPT* and *GRN* +mFTLD-CDR = 0 groups, there were a number of regions that reached a proportion of about 0.5, including the insula and medial temporal regions in *MAPT* carriers and the posterior temporal and parietal regions in *GRN* carriers. In the *C9orf72* +mFTLD-CDR = 0 group, the maximum proportion reached about 0.7, and this occurred in the thalamus on the right and the periinsular region on the left. Regions with proportions of about 0.6–0.8 were seen in the +mFTLD-CDR = 0.5 groups in all mutation types, located in the temporal region in *MAPT* carriers, the frontal region in *GRN* carriers, and in the thalamus and patchy regions in the frontal and temporal lobes in *C9orf72* carriers. The +mFTLD-CDR ≥ 1 *MAPT* group was the only one where the proportion reached 1, and this was in the temporal regions bilaterally. The +mFTLD-CDR ≥ 1 *GRN* and *C9orf72* groups both showed fairly diffuse regions of overlap including thalamus, bilateral insula, and medial parietal regions, with a few regions affecting nearly all participants in each group. Coordinates in the International Consortium for Brain Mapping space and anatomical labels for peak regions in each hemisphere in each group are provided in Supplementary Table 6 in the Supplementary Materials.

## 4. Discussion

The goal of this analysis was to characterize cognitive performance, behavioral ratings, and brain volumes in a large group of f-FTLD family members. In group comparisons, asymptomatic mutation carriers showed nearly identical scores on all clinical measures compared with noncarriers but reduced frontal and temporal lobe volumes. The group with mild/questionable impairment showed decreased story recall, word list recall, verbal fluency, processing speed, and set-shifting performance and impaired mood and self-monitoring. With development of dementia, all scores were abnormal compared with scores in less symptomatic groups. Looking at performance across individuals, the MoCA was frequently abnormal in all mutations, but this was also true in many noncarriers. The effects of *MAPT* mutations on brain volume and cognition were most consistent across individuals and stages, with naming impairment and temporal volume loss being present in a high proportion of carriers. Memory disorders were prominent in *GRN*, but *C9orf72* did not show a consistent pattern of impairment in the early stages, and both *GRN* and *C9orf72* showed lower levels of overlap in regional volume loss than *MAPT*.

These findings have important implications for research and therapy in f-FTLD, which is a critical context for testing treatments in the earliest phases of disease and also for testing whether treatments can prevent onset of symptoms. With regard to prevention, our finding that neuroimaging changes appear to precede clinical changes is consistent with multiple studies demonstrating brain volume loss and other brain imaging abnormalities in asymptomatic mutation carriers [21] and findings from a comprehensive study in a similar large cohort called the Genetic FTD Initiative (GENFI), which suggested that imaging findings precede symptom onset by more than 10 years [22]. These observations support the idea that imaging can serve as a leading indicator of clinical changes and that mutation carriers with imaging abnormalities will be important candidates for prevention studies. Additional work will be required to quantify the degree of abnormality that serves as an early marker, to quantify the timing until symptoms develop, and to assess the value of additional imaging techniques such as diffusion MRI and functional MRI [23].

Ideally, sensitivity for early detection of disease should improve if monitoring could be targeted at brain regions and clinical features that are most likely to be affected first in each mutation. In *MAPT*, we found very frequent involvement of the temporal lobe, which is also the region most associated with *MAPT* mutations in prior studies [24]. The consistency of this finding supports a strategy of monitoring early temporal lobe changes in *MAPT* carriers. However, the findings in our *GRN* and *C9orf72* cohorts suggest that focusing on a specific brain region in these groups would not capture early changes well in all individuals, although thalamic changes seemed to be fairly consistent in *C9orf72* carriers. Similarly, our clinical data do not point to one particular cognitive score that reliably marks early symptoms, even in *MAPT*. Although our finding that naming impairment is frequent in early *MAPT* carriers is similar to observations from GENFI [22], there were many asymptomatic and mildly/questionably symptomatic *MAPT* mutation carriers who showed impairment in other tasks but not in naming. Consistency across *GRN* and *C9orf72* mutation carriers appeared to be even lower, although abnormal trail making and fluency scores were relatively frequent in both groups, consistent with the frontoparietal involvement in both mutation types. This is in-line with prior observations that patients with FTLD mutations can present with a variety of clinical syndromes, even with the same mutation in the same family [1].

One approach for dealing with the heterogeneity in mutation carriers would be to track larger portions of the brain such as the frontal and temporal lobes. Similarly, one could use composite measures of cognition that represent function across multiple domains. The fact that the MoCA was one of the most frequently abnormal tests in carriers, even in the asymptomatic and mildly/questionably symptomatic groups, suggests that this might be a fruitful strategy. However, many noncarriers also showed abnormal performance on the MoCA, which suggests that relying on an arbitrary threshold to identify oncoming symptoms would limit the accuracy of the approach. Thus, additional longitudinal work will have to be done to empirically define performance thresholds that reliably predict development of functional changes. Another approach would be to use a multiple-predictor strategy to identify combinations of cognitive tests and behavioral measures from a battery such as the one used in this project to predict onset of symptoms. Such an approach could identify multiple patterns of impairment with predictive value and thus apply to a variety of clinical presentations. A similar approach can be used for brain imaging (see the article by Staffaroni et al. [25] in this issue for example).

These data illustrate the importance and promise of large longitudinal studies of f-FTLD such as LEFFTDS, GENFI, and similar efforts. While our findings reinforce the complexity and heterogeneity of FTLD, even in the context of disease-causing mutations, they suggest that early changes in imaging, cognitive performance, and behavioral ratings may be able to serve as early predictors of functional impairment and help to identify suitable candidates for prevention and early-stage treatment trials. As longitudinal data from these cohorts emerge, they will provide invaluable information about the earliest signs of FTLD and neurodegenerative disease in general.

## Supplementary Material

Supplement

## Figures and Tables

**Table 1:** Demographics, cognitive performance, and lobar volumes (cc’s) across groups

	−mFTLD-CDR = 0	+mFTLD-CDR = 0	+mFTLD-CDR = 0.5	+mFTLD-CDR ≥ 1
Demographics				
Number	102	103	43	72
Mean age*	47.53 [44.96, 50.1]^†,‡^	43.95 [41.22, 46.68]^‡,§^	55.44 [52.03, 58.85]^†^,§	60.17 [57.96, 62.38]^†,§^
M/F^¶^	44/58	49/54	22/21	30/42
Mean education	15.41 [14.93, 15.9]	15.78 [15.29, 16.27]	15.07 [14.26, 15.88]	15.42 [14.82, 16.01]
Cognitive performance (mean [95% CI]) values across groups				
MoCA*	27.23 [26.8, 27.66]	27.21 [26.78, 27.65]	25.12 [23.9, 26.34]^§^	17.48 [15.56, 19.41]^†,‡,§^
Memory				
Craft Immediate Recall*	22.5 [21.23, 23.77]	22.2 [20.95, 23.45]	19.61 [17.36, 21.86]	14.05 [11.83, 16.28]^†,‡,§^
Craft Delayed Recall*	20.74 [19.38, 22.1]	20.08 [18.79, 21.36]	16.71 [14.51, 18.91]^§^	11.15 [8.89, 13.4]^†,‡,§^
CVLT-Max Learning*	8.14 [7.94, 8.34]	8.3 [8.12, 8.48]	7.59 [7.03, 8.14]	5.81 [5.2, 6.41]^†,‡,§^
CVLT-Delay*	7.38 [7.05, 7.7]	7.24 [6.9, 7.58]	6.33 [5.46, 7.19]^§^	3.85 [3.03, 4.66]^†,‡,§^
Benson Delay*	12.91 [12.38, 13.44]	12.75 [12.26, 13.25]	11.05 [9.97, 12.12]^§^	7.97 [6.8, 9.13]^†,‡,§^
Visuospatial				
Benson Copy*	15.88 [15.64, 16.13]	15.76 [15.56, 15.97]	15.1 [14.28, 15.91]	13.97 [13.07, 14.87]^†,‡,§^
Language				
MINT*	30.03 [29.68, 30.38]	29.9 [29.44, 30.36]	28.86 [28, 29.71]	23.56 [21.62, 25.5]^†,‡,§^
Fluency Animals*	22.83 [21.73, 23.93]	23 [21.9, 24.1]	21.02 [19.22, 22.83]	12.28 [10.5, 14.07]^†,‡,§^
Fluency Vegetables*	14.44 [13.7, 15.18]	14.8 [14.06, 15.53]	12.17 [10.97, 13.37]^†,§^	8.39 [7.1, 9.68]^†,‡,§^
Fluency “L” words*	13.83 [13.01, 14.65]	14.17 [13.26, 15.09]	13.14 [11.63, 14.66]	6.27 [5.07, 7.46]^†,‡,§^
Fluency “F” words*	14.79 [13.88, 15.71]	15.67 [14.66, 16.68]	13.21 [12.05, 14.38]^†^	6.98 [5.81, 8.16]^†,‡,§^
Executive				
Digits Forward*	9.21 [8.72, 9.69]	8.78 [8.29, 9.26]	8.43 [7.78, 9.07]	6.03 [5.49, 6.58]^†,‡,§^
Digits Backward*	7.96 [7.48, 8.44]	8.21 [7.73, 8.7]	7.43 [6.73, 8.12]	4.31 [3.75, 4.86]^†,‡,§^
Trails A*	23.7 [22.17, 25.23]	23.6 [21.35, 25.86]	32.51 [28.68, 36.34]^§^	60.27 [50.6, 69.93]^†,‡,§^
Trails B*	59.32 [54.24, 64.41]	60.7 [56.31, 65.09]	83.56 [69.31, 97.8]^§^	154.69 [126.71, 182.67]^†,‡,§^
Behavior/mood				
NPI-Q*	1.02 [0.64, 1.41]	1.46 [0.9, 2.03]	5.78 [4, 7.55]^†,§^	9.19 [7.59, 10.79]^†,‡,§^
GDS*	1.81 [1.34, 2.28]	1.48 [1.1, 1.85]	3.07 [2.06, 4.08]^†,§^	2.73 [2.07, 3.39]^†,§^
BIS	17.05 [16.19, 17.92]	17.07 [16.27, 17.86]	17.51 [16.33, 18.7]	16.66 [15.6, 17.72]
RSMS*	48.11 [46.3, 49.92]	47.23 [45.13, 49.32]	39.51 [35.73, 43.3]^†,§^	20.66 [17.3, 24.01]^†,‡,§^
Motor	1.02 [0.64, 1.41]	1.46 [0.9, 2.03]	5.78 [4, 7.55]	9.19 [7.59, 10.79]
UPDRS*	0.1 [0, 0.2]	0.28 [0.04, 0.53]	2.24 [0.78, 3.69]	7.76 [4.36, 11.16]^†,‡,§^
PSPRS*	0.38 [0.09, 0.67]	0.37 [0.14, 0.6]	2.06 [0.83, 3.29]	8.9 [5.98, 11.81]^†,‡,§^
Brain volumes				
Frontal*	101.19 [98.65, 103.73]	98.17 [95.2, 101.14]^§^	90.7 [85.86, 95.53]^§^	72.08 [66.55, 77.6]^†,‡,§^
Temporal*	83.92 [81.99, 85.85]	81.82 [79.61, 84.02]^§^	76.38 [72.45, 80.31]^§^	61.01 [57.05, 64.98]^†,‡,§^

Abbreviations: MoCA, Montreal Cognitive Assessment; M, male; F, female; CI, confidence interval; MINT, Multilingual Naming Test; CVLT, California Verbal Learning Test; NPI-Q, Neuropsychiatric Investment Questionnaire; GDS, Geriatric Depression Scale; BIS, Behavioral Inhibition Scale; RSMS, Revised Self-Monitoring Scale; UPDRS, Unified Parkinson’s Disease Rating Scale; PSPRS, Progressive Supranuclear Palsy Rating Scale.

* *P* < .05 for effect of group in regression model.

† *P* < .05 compared with the +mFTLD-CDR0 group.

‡ *P* < .05 compared with the +mFTLD-CDR0.5 group.

§ P < .05 compared with the −mFTLD-CDR0 group.

¶ M/F comparisons used Chi-squared calculations.

## RESEARCH IN CONTEXT

1. Systematic review: The authors reviewed the literature using traditional sources (e.g., PubMed) and meeting abstracts and presentations.
2. Interpretation: Our results indicate that imaging abnormalities can serve as early indicators of oncoming functional deterioration in frontotemporal lobar degeneration. However, the specific brain regions and clinical abnormalities that herald the onset of functional change likely vary across individuals.
3. Future directions: The study lays the groundwork for future longitudinal studies to determine the timing between imaging and clinical changes and to define the best combination of imaging abnormalities and clinical measures for predicting functional changes.
