# Supplementary material for: Clinical and volumetric changes with increasing functional impairment in familial frontotemporal lobar degeneration
Source: Alzheimers Dement. Author manuscript; Available in PMC 2021 Jan 6. (PMC6988137; doi:10.1016/j.jalz.2019.08.196)
Supplement: Supplement [file NIHMS1067360-supplement-Supplement.docx]

**Supplemental Imaging Methods Description**

**Acquisition and Quality Control:** MRI’s for this study were acquired on 3 Tesla MRI Siemens, General Electric or Phillips scanners across 15 sites participating in ARTFL and LEFFTDS. The MRI acquisition for this multisite study was supervised by a central team at the Mayo Clinic in Rochester using the protocols established for the second cycle of the Alzheimer’s Disease Neuroimaging Initiative (ADNI-2). These have been extensively described elsewhere[1], but will be reviewed here briefly. ADNI has developed vendor and version specific acquisition protocols designed to maximize compatibility across scanner platforms. Scanners are qualified using phantom scans and requalified as needed with software upgrades or deviation from established quality parameters. The protocol uses formal quality control procedures, which begin with upload of images to a central location and download to a central site for review. Automated software is used to check tens of imaging parameters against the vendor/scanner specific protocol standards. In addition, scans are visually graded by a trained image analyst for quality and receive a score of 1-4 (1 = excellent quality, 4 = unusable) based on features that include movement artifact, completeness of anatomic coverage and medical findings. Scans acquired with incorrect protocol parameters or that have a quality score of 4 are excluded from analyses. This would include any images with strokes associated with encephalomalacia or any other lesions with visible effects on brain morphology, or other anatomical abnormalities (developmental, post-surgical) that would distort brain anatomy. The presence of subcortical white matter signal hyperintensity was not used as an inclusion/exclusion criterion because, although such changes may represent vascular disease, they are part of the spectrum of mutation-associated FTLD and also may represent direct effects of neurodegenerative pathology on the white matter[2-4]. The ADNI-2 protocol also implements post-acquisition bias field correction using the N3 algorithm, which reduces image non-uniformity and enhances the similarity of image segmentation results across acquisitions[5].

**Image Processing:** After bias correction, image segmentation was performed using the SPM12 (Wellcome Trust Center for Neuroimaging, London, UK, <http://www.fil.ion.ucl.ac.uk/spm>) unified segmentation algorithm[6]. A group template was generated from the segmented gray and white matter tissues and cerebrospinal fluid by non-linear registration using the Large Deformation Diffeomorphic Metric Mapping framework[7]. Native subject space gray and white matter were normalized to the group template space, followed by gray matter intensity modulation to account for image scaling required to match the template, and smoothing with a Gaussian kernel with 10~mm full width half maximum. Each step of segmentation and transformation from native space to the group template was carefully visually inspected for each image. Statistical analyses were performed in the study-specific group template. For visualization purposes and identification of peak coordinates for anatomical findings, linear and non-linear transformations between the group template space and International Consortium of Brain Mapping (ICBM[8]) was applied because most available templates are in ICBM space. Cortical volumes for the frontal and temporal lobes for each individual were also calculated by transforming the Desikan-Killiany brain parcellation atlas[9] into the study-specific brain space and summing all modulated gray matter within the 179 atlas-delineated regions for the frontal and temporal lobes.

| Table S1. Inferential statistics (t-score, p-value) for Group Comparisons Described in Table 1 | | | | | | | | | | | | |
| --- | --- | --- | --- | --- | --- | --- | --- | --- | --- | --- | --- | --- |
|  | +mFTLD-CDR=0 | | +mFTLD-CDR=0.5 | | | | +mFTLD-CDR≥1 | | | | | |
| Mutation Type | **Vs. -mFTLD-CDR=0** | | **Vs. -mFTLD-CDR=0** | | **Vs. +mFTLD-CDR=0** | | **Vs. -mFTLD-CDR=0** | | **Vs. +mFTLD-CDR=0** | | **Vs. +mFTLD-CDR=0.5** | |
|  | **t-score** | **p-value** | **t-score** | **p-value** | **t-score** | **p-value** | **t-**  **score** | **p-value** | **t-**  **score** | **p-**  **value** | **t-**  **score** | **p-**  **value** |
| Mean Age | -2.06 | 0.0405 | 3.51 | 0.0005 | 5.09 | <0.001 | 6.51 | <0.001 | 8.38 | <0.001 | 1.88 | 0.0615 |
| M/F | n/a | 0.6188 | n/a | 0.4816 | n/a | 0.8306 | n/a | 0.9700 | n/a | 0.5364 | n/a | 0.4258 |
| Mean Education | 1.06 | 0.2896 | -0.95 | 0.3436 | -1.72 | 0.0859 | -0.27 | 0.7908 | -1.13 | 0.2575 | 0.68, | 0.4956 |
| MoCA | -0.48 | 0.6342 | -2.16 | 0.0314 | -1.77 | 0.0783 | -12.0 | <0.001 | -12.15 | <0.001 | -9.09 | <0.001 |
| Craft Immediate Recall | -0.64 | 0.5246 | -1.93 | 0.0543 | -1.42 | 0.1562 | -6.58 | <0.001 | -5.85 | <0.001 | -3.84 | 0.0001 |
| Craft Delayed Recall | -1.02 | 0.3066 | -2.7 | 0.0073 | -1.89 | 0.0592 | -7.25 | <0.001 | -6.23 | <0.001 | -3.74 | 0.0002 |
| CVLT-Max Learning | 0.40 | 0.6860 | -1.4 | 0.1627 | -1.67 | 0.0968 | -8.19 | <0.001 | -8.21 | <0.001 | -5.83 | <0.001 |
| CVLT-Delay | -0.78 | 0.4492 | -2.14 | 0.0333 | -1.54 | 0.1250 | -8.53 | <0.001 | -7.66 | <0.001 | -5.42 | <0.001 |
| Benson Delay | -1.08 | 0.2798 | -2.26 | 0.0243 | -1.42 | 0.1562 | -7.61 | <0.001 | -6.52 | <0.001 | -4.44 | <0.001 |
| Benson Copy | -0.76 | 0.4516 | -1.53 | 0.1269 | -0.95 | 0.3450 | -4.66 | <0.001 | -3.91 | <0.001 | -2.57 | 0.0107 |
| MINT | -0.29 | 0.7721 | -1.89 | 0.0594 | -1.64 | 0.1019 | -9.99 | <0.001 | -9.41 | <0.001 | -6.81 | <0.001 |
| Fluency Animals | -0.24 | 0.8134 | -1.16 | 0.2468 | -0.96 | 0.3354 | -9.75 | <0.001 | -9.26 | <0.001 | -7.29 | <0.001 |
| Fluency Vegetables | 0.63 | 0.5276 | -2.85 | 0.0047 | -3.25 | 0.0013 | -8.6 | <0.001 | -8.79 | <0.001 | -4.81 | <0.001 |
| Fluency ‘L’ words | 0.08 | 0.9352 | -0.37 | 0.7101 | -0.43 | 0.6711 | -9.2 | <0.001 | -8.96 | <0.001 | -7.55 | <0.001 |
| Fluency ‘F’ words | 0.94 | 0.3465 | -1.44 | 0.1498 | -2.12 | 0.0358 | -9.25 | <0.001 | -9.66 | <0.001 | -6.59 | <0.001 |
| Digits Forward | -1.69 | 0.0927 | -1.26 | 0.2085 | 0.01 | 0.9955 | -7.02 | <0.001 | -5.48 | <0.001 | -4.88 | <0.001 |
| Digits Backward | 0.28 | 0.7813 | -0.67 | 0.5025 | -0.86 | 0.3885 | -8.34 | <0.001 | -8.29 | <0.001 | -6.55 | <0.001 |
| Trails A | 0.34 | 0.7320 | 2.19 | 0.0296 | 1.89 | 0.0604 | 10.56 | <0.001 | 9.95 | <0.001 | 7.15 | <0.001 |
| Trails B | 1.02 | 0.3072 | 2.15 | 0.0325 | 1.34 | 0.1802 | 10.42 | <0.001 | 9.43 | <0.001 | 7.51 | <0.001 |
| NPI-Q | 0.9 | 0.3667 | 6.04 | <0.001 | 5.27 | <0.001 | 11.44 | <0.001 | 10.38 | <0.001 | 4.05 | <0.001 |
| GDS | -0.9 | 0.3684 | 2.72 | 0.0069 | 3.32 | 0.0010 | 2.22 | 0.0270 | 2.83 | 0.005 | -0.57 | 0.5721 |
| BIS | 0.19 | 0.8531 | 0.71 | 0.4808 | 0.56 | 0.5774 | -0.51 | 0.6082 | -0.65 | 0.5153 | -1.10 | 0.2703 |
| RSMS | -0.78 | 0.4365 | -3.61 | 0.0004 | -2.96 | 0.0033 | -14.05 | <0.001 | -12.86 | <0.001 | -8.24 | <0.001 |
| UPDRS | 0.27 | 0.7894 | 1.79 | 0.0752 | 1.55 | 0.1211 | 7.0 | <0.001 | 6.57 | <0.001 | 4.3 | <0.001 |
| PSPRS | 0.22 | 0.8299 | 1.15 | 0.2521 | 0.97 | 0.3337 | 8.38 | <0.001 | 7.97 | <0.001 | 6.11 | <0.001 |
| Frontal | -2.23 | 0.0267 | -2.98 | 0.0033 | -1.19 | 0.2344 | -9.93 | <0.001 | -7.92 | <0.001 | -6.15 | <0.001 |
| Temporal | -2.37 | 0.0190 | -2.77 | 0.0062 | -0.89 | 0.3740 | -10.89 | <0.001 | -8.75 | <0.001 | -7.17, | <0.001 |

| Table S2. Impairments in Standardized Variables in **Non-carriers** | | |
| --- | --- | --- |
| Number in group | 102 | |
| Number, proportion with at least one variable abnormal | 70, 0.69 | |
|  | Number, proportion with selected variable abnormal | Number, proportion with **only this** selected variable abnormal |
| MoCA | **22, 0.22** | 3, 0.03 |
| Memory |  |  |
| Craft Immediate Recall | 12, 0.12 | 1, 0.01 |
| Craft Delayed Recall | 12, 0.12 | 1, 0.01 |
| Benson Delay | 11, 0.11 | 3, 0.03 |
| Visuospatial |  |  |
| Benson Copy | 9, 0.09 | 2, 0.02 |
| Language | 9, 0.09 |  |
| **MINT** | **20, 0.2** | **8, 0.08** |
| Fluency Animals | 12, 0.12 | 2, 0.02 |
| Fluency Vegetables | 12, 0.12 | 1, 0.01 |
| Fluency ‘L’ words | 10, 0.1 | 1, 0.01 |
| Fluency ‘F’ words | 10, 0.1 | 0 |
| Executive |  |  |
| Digits Forward | 3, 0.03 | 1, 0.01 |
| Digits Backward | 10, 0.1 | 2, 0.02 |
| Trails A | 12, 0.12 | 3, 0.03 |
| Trails B | 13, 0.13 | 2, 0.02 |

| Table S3. Impairments in Standardized Variables in ***MAPT* carriers** | | | | | | |
| --- | --- | --- | --- | --- | --- | --- |
|  | +mFTLD-CDR=0 | | +mFTLD-CDR=0.5 | | +mFTLD-CDR≥1 | |
| Number in group* | 35 | | 12 | | 20 (16 with less than 2 tests missing) | |
| Number, proportion with at least one variable abnormal | 24, 0.69 | | 10, 0.83 | | 16, 1.0 | |
|  | Number, proportion with selected variable abnormal | Number, proportion with **only this** selected variable abnormal | Number, proportion with selected variable abnormal | Number, proportion with **only this** selected variable abnormal | Number, proportion with selected variable abnormal | Number, proportion with **only this** selected variable abnormal |
| MoCA | **10, 0.29** | 0 | **7, 0.58** | 2, 0.17 | **13, 0.81** | 0 |
| Memory |  |  |  |  |  |  |
| Craft Immediate Recall | 5, 0.15 | 0 | 3, 0.25 | 0 | 7, 0.44 | 0 |
| Craft Delayed Recall | 3, 0.09 | 0 | 2, 0.25 | 0 | 8, 0.5 | 0 |
| Benson Delay | 3, 0.09 | 0 | 3, 0.25 | 0 | 8, 0.5 | 0 |
| Visuospatial |  |  |  |  |  |  |
| Benson Copy | 7, 0.21 | 1, 0.04 | 1, 0.08 | 0 | 4, 0.25 | 0 |
| Language |  |  |  |  |  |  |
| MINT | **12, 0.32** | 3, 0.13 | **7, 0.58** | 1, 0.08 | **13, 0.81** | 0 |
| Fluency Animals | 4, 0.11 | 0 | 2, 0.17 | 0 | **14, 0.88** | 0 |
| Fluency Vegetables | 4, 0.11 | 0 | **5, 0.42** | 1, 0.08 | 9, 0.56 | 0 |
| Fluency ‘L’ words | 4, 0.11 | 0 | 1, 0.08 | 0 | 9, 0.56 | 0 |
| Fluency ‘F’ words | 3, 0.09 | 0 | 2, 0.17 | 0 | 9, 0.56 | 0 |
| Executive |  |  |  |  |  |  |
| Digits Forward | 2, 0.06 | 0 | 0 | 0 | 5, 0.31 | 0 |
| Digits Backward | 3, 0.09 | 0 | 1, 0.08 | 0 | 6, 0.38 | 0 |
| Trails A | 3, 0.09 | 1, 0.04 | 2, 0.17 | 0 | 11, 0.69 | 0 |
| Trails B | 4, 0.11 | 0 | 1, 0.08 | 0 | 8, 0.5 | 0 |
| * = To allow more meaningful comparison across subjects, participants with more than two missing scores were left out | | | | | | |

| Table S4. Impairments in Standardized Variables in ***GRN* carriers** | | | | | | |
| --- | --- | --- | --- | --- | --- | --- |
|  | +mFTLD-CDR=0 | | +mFTLD-CDR=0.5 | | +mFTLD-CDR≥1 | |
| Number in group* | 28 | | 12 | | 16 (14 with less than 2 tests missing) | |
| Number, proportion with at least one variable abnormal | 20, 0.71 | | 9, 0.75 | | 14, 1.0 | |
|  | Number, proportion with selected variable abnormal | Number, proportion with **only this** selected variable abnormal | Number, proportion with selected variable abnormal | Number, proportion with **only this** selected variable abnormal | Number, proportion with selected variable abnormal | Number, proportion with **only this** selected variable abnormal |
| MoCA | **5, 0.18** | 0 | **4, 0.33** | 1, 0.08 | **12, 0.86** | 0 |
| Memory |  |  |  |  |  |  |
| Craft Immediate Recall | **5, 0.18** | 0 | 2, 0.17 | 0 | 6, 0.43 | 0 |
| Craft Delayed Recall | **6, 0.21** | 1 | **3, 0.25** | 0 | 7, 0.5 | 0 |
| Benson Delay | 4, 0.14 | 0 | 0 | 0 | 8, 0.57 | 0 |
| Visuospatial |  |  |  |  |  |  |
| Benson Copy | 1, 0.04 | 1, 0.04 | 2, 0.17 | 0 | 5, 0.36 | 0 |
| Language | 1, 0.04 |  | 2, 0.22 |  | 4, 0.44 |  |
| MINT | 0, 0 | 3, 0.11 | 2, 0.17 | 1, 0.08 | 5, 0.36 | 0 |
| Fluency Animals | 3, 0.11 | 1, 0.04 | 1, 0.08 | 1, 0.08 | 8, 0.57 | 0 |
| Fluency Vegetables | 4, 0.14 | 1, 0.04 | 3, 0.25 | 0 | 8, 0.57 | 0 |
| Fluency ‘L’ words | 2, 0.07 | 0 | 0, 0 | 0 | **9, 0.64** | 0 |
| Fluency ‘F’ words | 3, 0.11 | 0 | **3, 0.25** | 0 | 8, 0.57 | 0 |
| Executive |  |  |  |  |  |  |
| Digits Forward | **5, 0.18** | 1, 0.04 | 0, 0 | 0 | 3, 0.21 | 0 |
| Digits Backward | 4, 0.14 | 0 | 2, 0.18 | 0 | **9, 0.64** | 0 |
| Trails A | 1, 0.04 | 1, 0.04 | **3, 0.25** | 1, 0.08 | **9, 0.64** | 0 |
| Trails B | 4, 0.14 | 2, 0.08 | **3, 0.25** | 0 | 7, 0.5 | 0 |
| * = To allow more meaningful comparison across subjects, participants with more than two missing scores were left out | | | | | | |

| Table S5. Impairments in Standardized Variables in ***C9orf72* carriers** | | | | | | |
| --- | --- | --- | --- | --- | --- | --- |
|  | +mFTLD-CDR=0 | | +mFTLD-CDR=0.5 | | +mFTLD-CDR≥1 | |
| Number in group* | 40 | | 19 | | 36 (35 with less than 2 tests missing) | |
| Number, proportion with at least one variable abnormal | 29, 0.73 | | 17, 0.89 | | 34, 0.97 | |
|  | Number, proportion with selected variable abnormal | Number, proportion with **only this** selected variable abnormal | Number, proportion with selected variable abnormal | Number, proportion with **only this** selected variable abnormal | Number, proportion with selected variable abnormal | Number, proportion with **only this** selected variable abnormal |
| MoCA | 6, 0.15 | 0 | **8, 0.42** | 0 | **27, 0.77** | 0 |
| Memory |  |  |  |  |  |  |
| Craft Immediate Recall | 5, 0.13 | 0 | 3, 0.16 | 0 | 16, 0.46 | 0 |
| Craft Delayed Recall | 5, 0.13 | 1, 0.03 | 2, 0.11 | 0 | 11, 0.31 | 0 |
| Benson Delay | 7, 0.18 | 2, 0.06 | 4, 0.21 | 1, 0.05 | 11, 0.31 | 0 |
| Visuospatial |  |  |  |  |  |  |
| Benson Copy | 2, 0.05 | 0 | 5, 0.26 | 0 | 13, 0.37 | 0 |
| Language | 1, 0.04 |  | 2, 0.22 |  | 4, 0.44 |  |
| MINT | **9, 0.23** | 1, 0.03 | 4, 0.21 | 0 | 22, 0.63 | 0 |
| Fluency Animals | 5, 0.13 | 1, 0.03 | 5, 0.26 | 0 | **24, 0.69** | 0 |
| Fluency Vegetables | 4, 0.1 | 1, 0.03 | 3, 0.16 | 0 | 18, 0.51 | 0 |
| Fluency ‘L’ words | 2, 0.05 | 0 | 3, 0.16 | 0 | 23, 0.66 | 0 |
| Fluency ‘F’ words | 7, 0.18 | 1, 0.03 | 1, 0.05 | 0 | 21, 0.6 | 0 |
| Executive |  |  |  |  |  |  |
| Digits Forward | 3, 0.08 | 1, 0.03 | 2, 0.1 | 0 | 10, 0.29 | 0 |
| Digits Backward | 1, 0.03 | 1, 0.03 | 1, 0.05 | 0 | 19, 0.54 | 0 |
| Trails A | 5, 0.13 | 3, 0.09 | **11, 0.58** | 2, 0.1 | 22, 0.63 | 0 |
| Trails B | 8, 0.2 | 1, 0.03 | 8, 0.42 | 0 | 15, 0.43 | 0 |
| * = To allow more meaningful comparison across subjects, participants with more than two missing scores were left out | | | | | | |

| Table S6. Proportion, Coordinates and Anatomical Labels for Peak Regions of Overlap in Atrophy Ccross Severity Groups in Mutation Carriers | | | | | | | | | | |
| --- | --- | --- | --- | --- | --- | --- | --- | --- | --- | --- |
|  |  | +mFTLD-CDR=0 | | | +mFTLD-CDR=0.5 | | | +mFTLD-CDR≥1 | | |
|  | **Side** | **Prop** | **Coord** | **Region** | **Prop** | **Coord** | **Region** | **Prop** | **Coord** | **Region** |
| *MAPT* | *L* | 0.48 | -30,1,4 | Insula | 0.77 | -45,5,38 | Middle Temporal | 1 | -26,-6,-18 | Amygdala |
|  | *R* | 0.5 | 49,-10,33 | Inferior Temporal | 0.74 | 32,-24,-9 | Hippocampus | 0.99 | 30,9,-29 | Temporal Pole |
| *GRN* | *L* | 0.5 | -29,-73,-12 | Fusiform | 0.6 | -6,24,66 | Superior Frontal | 0.9 | -36,8,11 | Insula |
|  | *R* | 0.37 | 42,-24,21 | Supra-marginal | 0.57 | 0,47,-24 | Medial Orbitofrontal | 0.89 | 8,11,42 | Superior Frontal |
| *C9orf72* | *L* | 0.67 | -42,-24,3 | Transverse Temporal | 0.78 | -18,-27,2 | Thalamus | 0.95 | -5,-75,29 | Cuneus |
|  | *R* | 0.59 | 21,-29,5 | Thalamus | 0..79 | 18,-25,5 | Thalamus | 0.97 | 15,-23,2 | Thalamus |

**References**

[1] Jack CR, Jr., Barnes J, Bernstein MA, Borowski BJ, Brewer J, Clegg S, et al. Magnetic resonance imaging in Alzheimer's Disease Neuroimaging Initiative 2. Alzheimers Dement. 2015;11:740-56.

[2] Ameur F, Colliot O, Caroppo P, Stroer S, Dormont D, Brice A, et al. White matter lesions in FTLD: distinct phenotypes characterize GRN and C9ORF72 mutations. Neurol Genet. 2016;2:e47.

[3] Paternico D, Premi E, Gazzina S, Cosseddu M, Alberici A, Archetti S, et al. White matter hyperintensities characterize monogenic frontotemporal dementia with granulin mutations. Neurobiol Aging. 2016;38:176-80.

[4] Tacik P, Sanchez-Contreras M, DeTure M, Murray ME, Rademakers R, Ross OA, et al. Clinicopathologic heterogeneity in frontotemporal dementia and parkinsonism linked to chromosome 17 (FTDP-17) due to microtubule-associated protein tau (MAPT) p.P301L mutation, including a patient with globular glial tauopathy. Neuropathol Appl Neurobiol. 2017;43:200-14.

[5] Boyes RG, Gunter JL, Frost C, Janke AL, Yeatman T, Hill DL, et al. Intensity non-uniformity correction using N3 on 3-T scanners with multichannel phased array coils. Neuroimage. 2008;39:1752-62.

[6] Ashburner J, Friston KJ. Unified segmentation. Neuroimage. 2005;26:839-51.

[7] Ashburner J, Friston KJ. Diffeomorphic registration using geodesic shooting and Gauss-Newton optimisation. Neuroimage. 2011;55:954-67.

[8] Fonov V, Evans AC, Botteron K, Almli CR, McKinstry RC, Collins DL, et al. Unbiased average age-appropriate atlases for pediatric studies. Neuroimage. 2011;54:313-27.

[9] Desikan RS, Segonne F, Fischl B, Quinn BT, Dickerson BC, Blacker D, et al. An automated labeling system for subdividing the human cerebral cortex on MRI scans into gyral based regions of interest. Neuroimage. 2006;31:968-80.
